# Advancing ASMS with LC-MS/MS for the discovery of novel PDCL2 ligands from DNA-encoded chemical library selections

**DOI:** 10.1111/andr.13309

**Published:** 2022-11-02

**Authors:** Qiuji Ye, Hassane Belabed, Yong Wang, Zhifeng Yu, Murugesan Palaniappan, Jian-Yuan Li, Stacey A. Kalovidouris, Kevin R. MacKenzie, Mingxing Teng, Damian W. Young, Yoshitaka Fujihara, Martin M. Matzuk

**Affiliations:** 1 Center for Drug Discovery, Department of Pathology & Immunology, Baylor College of Medicine, Houston, Texas, USA; 2 Department of Pharmacology and Chemical Biology, Baylor College of Medicine, Houston, Texas, USA; 3 National Cerebral and Cardiovascular Center, Suita, Japan

**Keywords:** affinity selection mass spectrometry, DNA-encoded chemical libraries, medicinal chemistry

## Abstract

**Background:**

A safe, effective, and reversible nonhormonal male contraceptive drug is greatly needed for male contraception as well as for circumventing the side effects of female hormonal contraceptives. Phosducin-like 2 (PDCL2) is a testis-specific phosphoprotein in mice and humans. We recently found that male PDCL2 knockout mice are sterile due to globozoospermia caused by impaired sperm head formation, indicating that PDCL2 is a potential target for male contraception. Herein, our study for the first time developed a biophysical assay for PDCL2 allowing us to screen a series of small molecules, to study structure-activity relationships, and to discover two PDCL2 binders with novel chemical structure.

**Objective:**

To identify a PDCL2 ligand for therapeutic male contraception, we performed DNA-encoded chemical library (DECL) screening and off-DNA hit validation using a unique affinity selection mass spectrometry (ASMS) biophysical profiling strategy.

**Materials and methods:**

We employed the screening process of DECL, which contains billions of chemically unique DNA-barcoded compounds generated through individual sequences of reactions and different combinations of functionalized building blocks. The structures of the PDCL2 binders are proposed based on the sequencing analysis of the DNA barcode attached to each individual DECL compound. The proposed structure is synthesized through multistep reactions. To confirm and determine binding affinity between the DECL identified molecules and PDCL2, we developed an ASMS assay that incorporates liquid chromatography with tandem mass spectrometry (LC-MS/MS).

**Results:**

After a screening process of PDCL2 with DECLs containing >440 billion compounds, we identified a series of hits. The selected compounds were synthesized as off-DNA small molecules, characterized by spectroscopy data, and subjected to our ASMS/LC-MS/MS binding assay. By this assay, we discovered two novel compounds, which showed good binding affinity for PDCL2 in comparison to other molecules generated in our laboratory and which were further confirmed by a thermal shift assay.

**Discussion and conclusion and relevance:**

With the ASMS/LC-MS/MS assay developed in this paper, we successfully discovered a PDCL2 ligand that warrants further development as a male contraceptive.

## 1 ∣ INTRODUCTION

Phosducin-like 2 (PDCL2) is encoded by the phosducin-II subgroup (PhLP2) of the phosducin (Pdc) gene family.^1^ It has been found that PDCL2 forms a complex with heat shock protein Hsp90,^2^ functions as chaperone, and is essential for cell growth in unicellular organisms.^3,4,5^ More importantly, PDCL2 is a testis-specific phosphoprotein in mice and humans.^6,7^ Previously, we discovered that CRISPR/Cas9-mediated PDCL2 mutant male mice are sterile due to globozoospermia caused by impaired sperm head formation (accompanying paper submitted to Andrology). This finding motivated our search for small molecule PDCL2 ligands that could disrupt sperm head formation and thereby be useful clinical candidates for nonhormonal contraception in men.

**Figure 1.**
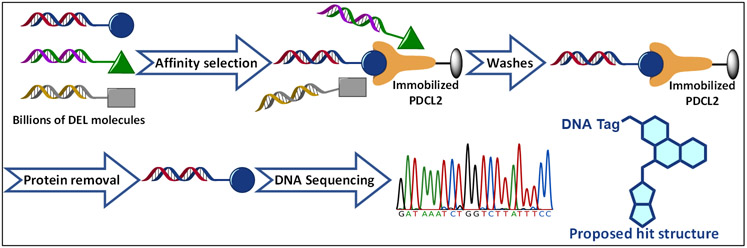
Workflow of DECL selection assay of PDCL2 and billions of DECL molecules. The DECL compounds are incubated with immobilized PDCL2, treated with selection washes, processed by protein removal, and subjected to DNA sequencing. Compounds with high sequencing counts are selected as hit molecules.

To identify and discover a small molecule ligand for PDCL2, we employed the screening of DNA-encoded chemical libraries (DECLs), which contain billions of chemically unique DNA-barcoded compounds. These DECLs are generated through individual sequences of reactions and different combinations of functionalized building blocks (Figure 1).^8-11^ To screen the library compounds, an affinity selection experiment to PDCL2 was applied to the library pool. The affinity selection is a binding-based screen that retains those DECL molecules that bind with reasonably high affinity to PDCL2 that is immobilized on a solid support while removing nonbinding DECL molecules by washing. The structures of the PDCL2 binders are proposed based on sequencing analysis^12^ of the DNA barcodes that are covalently linked to each individual DECL compound (Figure 1). Although DECL screening analysis can generate several chemical series enriched with certain structure-enrichment relationships (SER), such statistical results cannot exactly represent the binding activity of the corresponding chemical structure. In addition, because PDCL2 is not an active enzyme, traditional enzyme assays are not available for screening and measuring small molecule inhibition with PDCL2. To overcome this limitation, we developed a unique affinity selection mass spectrometry (ASMS) strategy to evaluate the PDCL2 binding affinity of small molecules identified by DECL.

**Figure 2.**
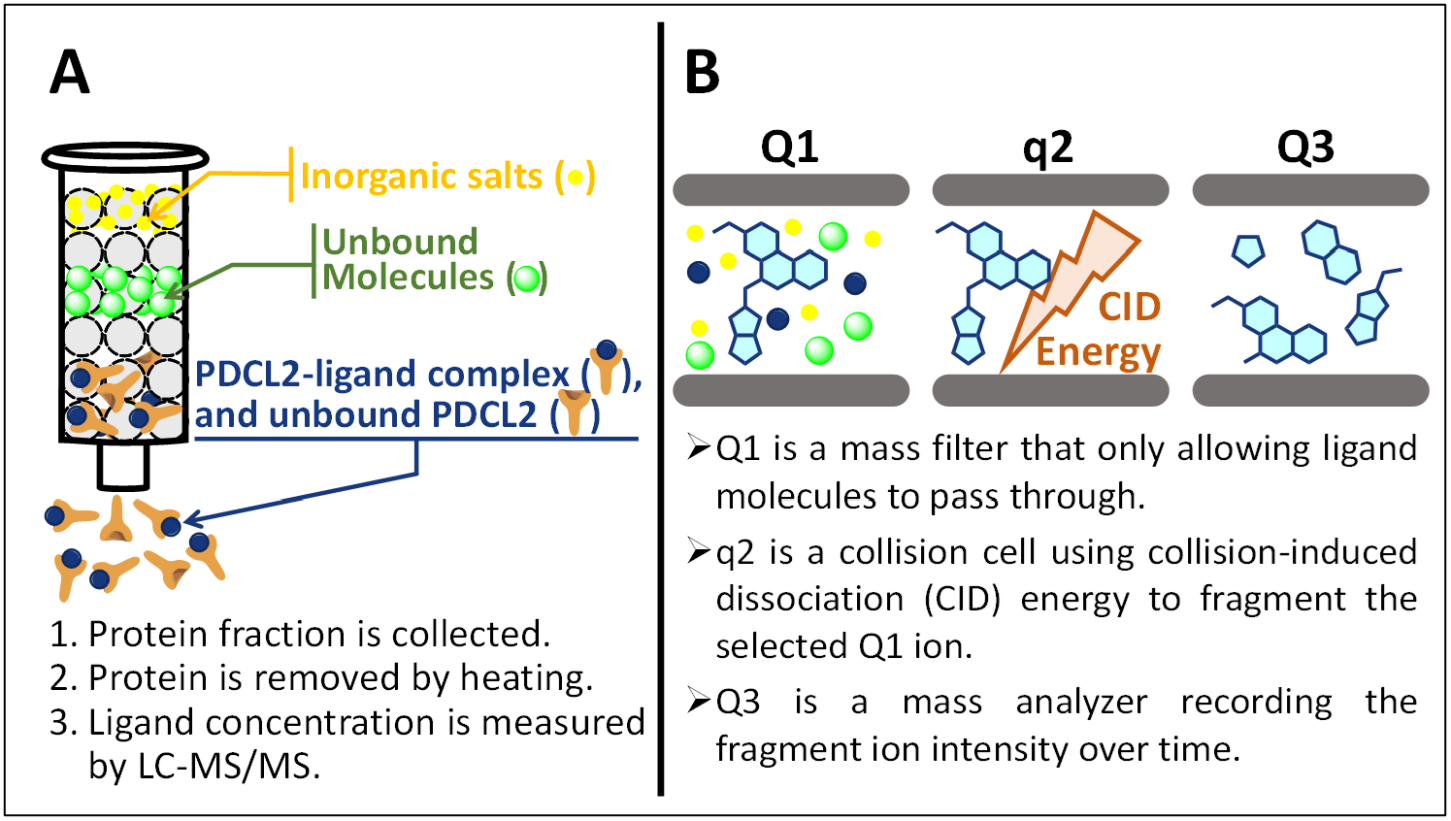
Mechanism of ASMS assay developed based on LC-MS/MS technique. Figure 2A shows the protein and ligand-protein complex are separated from salts and small organic molecule impurities. Figure 2B shows the function of each quadrupole in the LC-MS/MS system, which allows the highly sensitive and reproducible measurement of the ligand concentration.

The ASMS assay^13-15^ developed in this paper is based on a liquid chromatography with tandem mass spectrometry (LC-MS/MS) technique (Figure 2). To the best of our knowledge, a systematic method development to incorporate LC-MS/MS in a DECL/ASMS approach for protein ligand discovery has not been reported. By taking advantage of the well-developed LC-MS/MS technique, ligand signal was detected with high selectivity and sensitivity, which allows the detection and quantification of small molecule with high accuracy at picomolar concentration. The basic workflow of the ASMS binding affinity assay starts with incubating the small molecule hits with PDCL2 in buffer solution. For an active compound, the ligand-PDCL2 complex was eluted and collected after passing through a size-exclusion column (SEC), and the amount of the bound ligands was detected by LC-MS/MS in the following procedures. For nonbinding compounds, the free small molecule is adsorbed by the SEC column material, leaving the PDCL2 protein alone to be eluted out from the SEC column (Figure 2A). The absence of small molecule in the nonbinder SEC eluant will show zero signal intensity in LC-MS/MS data. The collected eluant containing either ligand-PDCL2 complex (binder compound presence) or PDCL2 alone (nonbinder compound presence) was then denatured by heating to allow any small molecule binder, in the case when a ligand-PDCL2 complex is formed, to be released and detected by mass spectrometry. Importantly, the ASMS method extensively reproduces the exact binding conditions as the DECL screening condition, making it straightforward to compare the binding affinity between the on-DNA compound and the corresponding off-DNA compound. Moreover, the binding affinity in ASMS is evaluated by the actual amount of molecules that bound to the protein, rendering an unambiguous result for structure-activity relationship (SAR) analysis and studies.

Samples obtained from SEC contain organic buffer molecules, organic detergent molecules, denatured protein residues, and other impurities, which could have an overlapping signal (retention time and mass) with the ligand signal and interfere with the quantification results. Rather than using conventional LC/MS to analyze the complex milieu collected from SEC, we applied LC-MS/MS, a highly sensitive and reproducible technique,^16-19^ to quantify the ligand and evaluate the binding affinity of the small molecules. To decrease the background noise and improve the signal intensity of the small molecule ligand, we developed a selected reaction monitoring mass spectrometry method with a triple quadrupole (QqQ) MS instrument operating in the product-ion scan mode (Figure 2B). In the QqQ instrument, the first quadrupole (Q1, Figure 2B) is set to remove background ions and isolate the ligand precursor ion, which is then fragmented in the second quadrupole (q2, Figure 2B). The generated fragment ions are then transferred to the third quadrupole (Q3, Figure 2B) for mass analysis and signal intensity measurement (Figure 2B). We measure the binding affinity based on the signal intensity detected by the LC-MS/MS system. This paper describes our efforts in identifying a novel PDCL2 ligand from DECLs and improvement of the ASMS assay using a LC-MS/MS technique.

## 2 ∣ RESULTS AND DISCUSSION

### 2.1 ∣ DECL screening results with PDCL2

To identify small molecules that bind to PDCL2, DECLs (from HitGen) cumulatively containing >440 billion compounds were pooled together for parallel screening of PDCL2 at 5 *μ*M, 1 *μ*M, and 0.2 *μ*M concentrations. Blank control affinity selection was performed in parallel without protein to identify any nonspecific bead binders. Bioinformatics sequencing analysis of the amplified DNA barcodes was processed and presented as count number to approximate the relative binding affinity of on-DNA molecules to the PDCL2 protein during the screening process. The sequencing data identified a series of bis-substituted pyrimidine candidates enriched with good SER from one of HitGen’s DECL libraries (DEL 1113, Table 1).^12,20^ As shown in Table 1, the selection counts in this series of hit candidates positively relate to the PDCL2 concentration in the selection screenings indicating a reliable DEL screening result. The enriched hit series contains a pyrimidine core from the Cycle 1 (C1) building block (blue, Table 1), a heterocyclic Cycle 2 building block (C2, red, Table 1) with a primary amine group enabling C-N bond attachment to the C1 pyrimidine core, and a phenyl borylating reagent as the Cycle 3 building block (C3, gray, Table 1) for Suzuki coupling with the C1 core. Based on the SER analysis, we selected three candidates shown in Table 1 to investigate further by performing their off-DNA organic syntheses.

### 2.2 ∣ Validation and synthesis of PDCL2 selection hits

**Figure 3.**
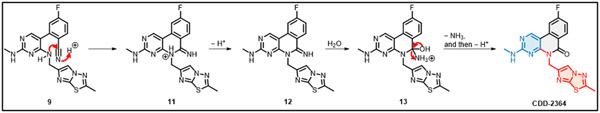
Reaction mechanism for the formation of **CDD-2364** from **9**. Once **9** is generated from the Suzuki reaction, the amine nitrogen atom attacks the nitrile carbon 5-bond away and forms a six-membered ring with an imine structure (**11**). **11** underwent a deprotonation process to form **12**, which is hydrolyzed into **CDD-2364** through **13**.

**Scheme 1.**
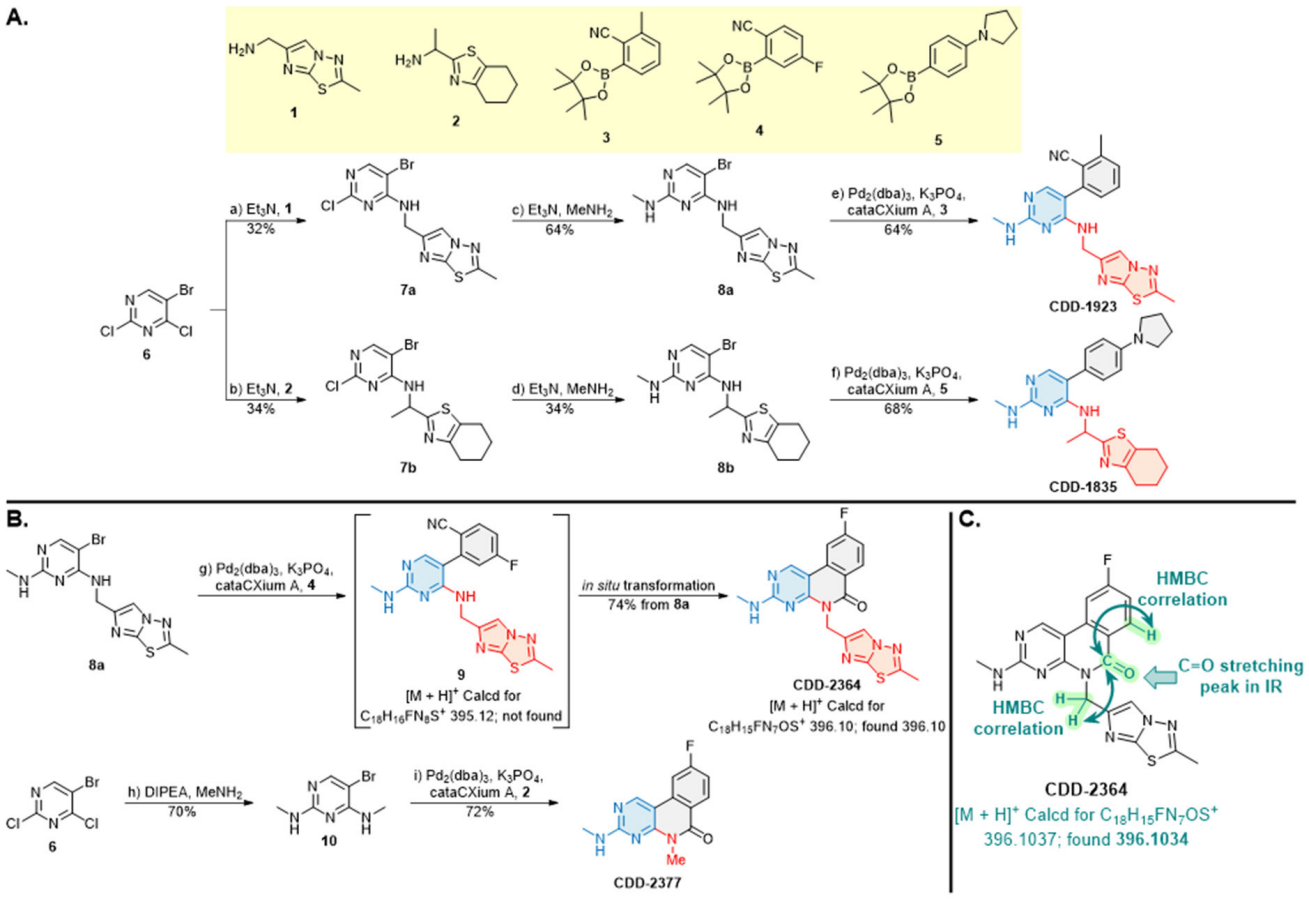
Synthesis of CDD-1923, 1835, 2364, and 2377^a^ ^*a*^Reagents and conditions: (a) Et_3_N (3.0 equiv.), **1** (1.3 equiv.), MeCN, reflux, 18 h, 32%; (b) Et_3_N (2.0 equiv.), **2** (1.3 equiv.), MeCN, 23 °C, 18 h, 34%; (c) Et_3_N (2.0 equiv.), MeNH_2_ (3.0 equiv.), EtOH, 80 °C, 18 h, 64%; (d) Et_3_N (2.0 equiv.), MeNH_2_ (3.0 equiv.), EtOH, 80 °C, 18 h, 34%; (e) Pd_2_(dba)_3_ (0.05 equiv.), cataCXium A (0.1 equiv.), K_3_PO_4_ (2.0 equiv.), **3** (1.2 equiv.), dioxane:H_2_O (5:1, *v*/*v*), MW (110 °C), 30 min, 64%; (f) Pd_2_(dba)_3_ (0.05 equiv.), cataCXium A (0.1 equiv.), K_3_PO_4_ (2.0 equiv.), **5** (1.2 equiv.), dioxane:H_2_O (5:1, *v*/*v*), MW (110 °C), 30 min, 68%; (g) Pd_2_(dba)_3_ (0.05 equiv.), cataCXium A (0.1 equiv.), K_3_PO_4_ (2.0 equiv.), **4** (1.2 equiv.), dioxane:H_2_O (5:1, *v*/*v*), MW (110 °C), 30 min, 74% from **8a**; (h) MeNH_2_•HCl (6.0 equiv.), DIPEA:DMA (2:1, *v*/*v*), 110 °C, 18 h, 70%; (i) Pd_2_(dba)_3_ (0.05 equiv.), cataCXium A (0.1 equiv.), K_3_PO_4_ (2.0 equiv.), **2** (1.2 equiv.), dioxane:H_2_O (5:1, *v*/*v*), MW (110 °C), 30 min, 72%. Pd_2_(dba)_3_ = tris(dibenzylideneacetone)dipalladium(0), cataCXium A = di(1-adamantyl)-n-butylphosphine, DIPEA = N,N-diisopropylethylamine, DMA = N,N-dimethylacetamide, HMBC = heteronuclear multiple bond correlation, IR = infrared spectroscopy. The color in the CDD compound structure represents functional groups derived from C1, C2, and C3 building block respectively (C1 in blue, C2 in red, and C3 in black).

Candidate hit molecules were synthesized by truncating the DNA barcode linkage down to a methyl amide (Table 1 and Scheme 1). The synthesis route was redesigned from the on-DNA library synthesis as shown in Scheme 1. To prepare the tri-synthon parent compounds (**CDD-1923**, **CDD-1835**, and **CDD-2364**), we commenced with the C1 building block bromo-dichloropyrimidine (**6**) and installed the amine C2 building block (**1**, or **2**) via an S_N_Ar reaction under basic conditions to produce the intermediates **7a** and **7b,** respectively. The chloro group in **7a** and **7b** was further eliminated and substituted with methyl amine to yield the bis-amine intermediates (**8a** and **8b**). The methyl amine group is a surrogate for the DNA attachment point of the DECL on-DNA compounds and maintains the binding affinity. The bromo intermediates (**8a** and **8b**) were further reacted with the borylated reagents (**3** and **5**) using Suzuki–Miyaura reaction conditions to afford the final products **CDD-1923** and **CDD-1835**, respectively. During the transformation of **8a** to **9**, the resulting mass ion peak for the product in the LC-MS analysis was 1 mass units greater than the expected (m/z was determined to be 396.1 instead of 395.1 for **9**). Extensive characterization of the product by ^1^H NMR, ^13^C NMR, HSQC, HMBC, HRMS, and IR analysis confirmed it to be phenanthridinone **CDD-2364** (Scheme 1C and shown in Supporting Information). This is rationalized on grounds that product **9** is initially formed but undergoes subsequent reactions. The nitrile group in **9** is activated by the presence of the *ortho*-pyrimidine and *para*-fluoro substituents—this favors its intramolecular attack by the amine to form an amidine intermediate, which hydrolyzes into the phenanthridinone product (Figure 3). Based on this observation and literature precedent,^21-30^ we believe that the same reaction mechanism occurs during the DECL synthesis to generate the phenanthridinone structure as the major product on DNA. Inspired by the unexpected phenanthridinone formation, we prepared **CDD-2377** (Scheme 1B) as the C2 truncated version of **CDD-2364** in a similar manner for structural activity relationship studies.

### 2.3 ∣ ASMS results

**Figure 4.**
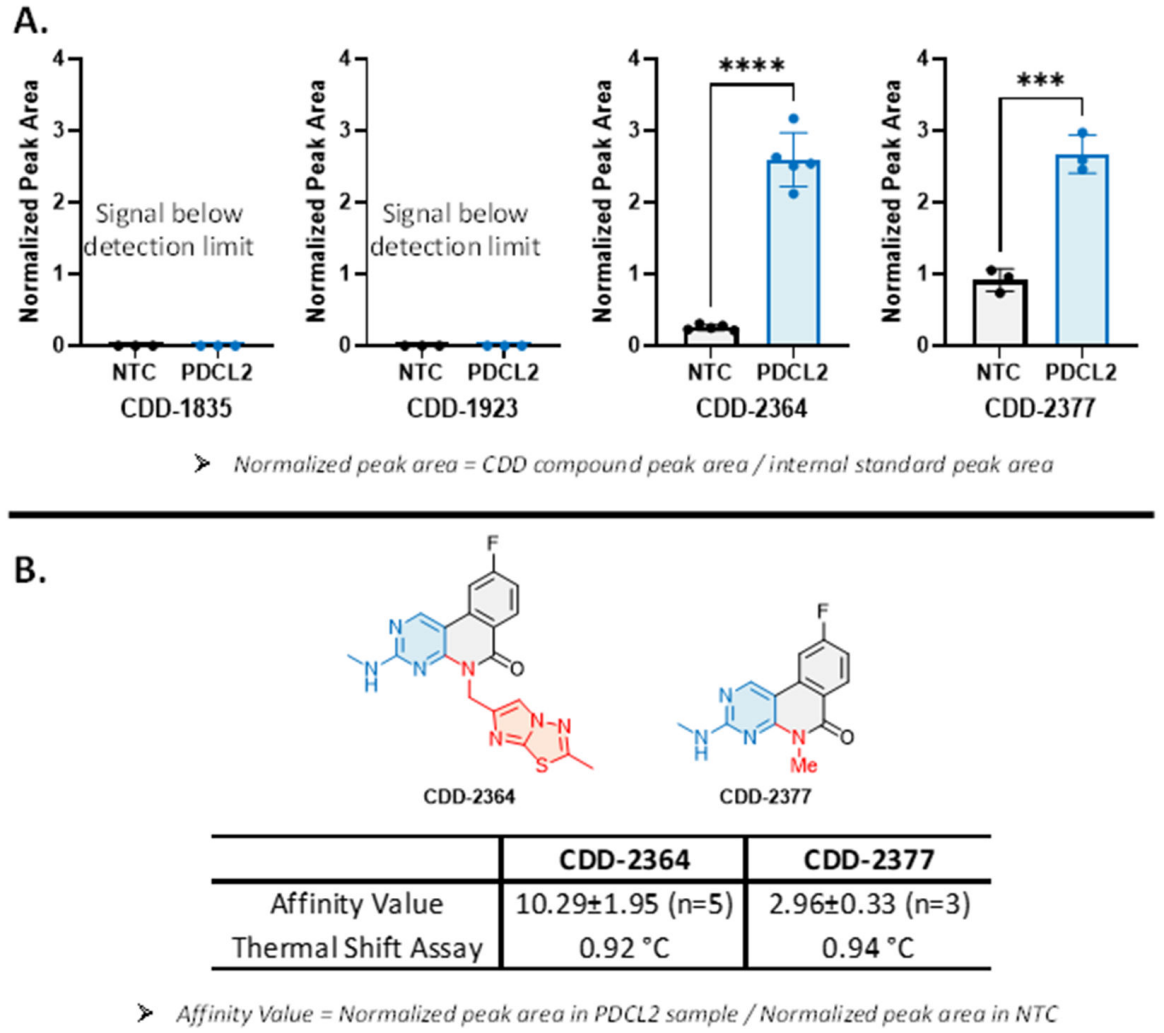
Binding assay results of **CDD-1835**, **CDD-1923**, **CDD-2364**, and **CDD-2377**. Panel A shows the normalized peak area of NTC sample, and PDCL2 incubated sample for each CDD compounds. Affinity enrichment was only observed with **CDD-2364** and **CDD-2377** compounds. Panel B calculates the affinity value based on the equation shown at the bottom of the figure. Panel B also shows the thermal shift assay (TSA) results of **CDD-2364** and **CDD-2377**, and the TSA data is consistent with the ASMS results.

The binding affinity of each CDD compounds to PDCL2 was determined by the ASMS assay. The ASMS assay for each CDD compound included a nontarget control (NTC) sample and a PCDL2 incubated sample. The NTC sample served as a blank control and was prepared by the exact same procedure as the PDCL2 incubated sample without adding the PDCL2 aliquot. Both the NTC sample solution and the PDCL2 containing sample solution were incubated at 25°C for 45 min, passed through the SEC column, processed by heat elution condition, and centrifuged to obtain the supernatant for LC-MS/MS experiment. The difference in the CDD compound peak area between the NTC sample and the PDCL2 incubated sample reveals the binding affinity of the CDD compound. The affinity value was calculated based on LC-MS/MS peak areas using Equations 1 and 2 (also shown in Figure 4A,B).^31^ The peak area of the CDD compound in the PDCL2 incubated sample and the NTC sample was first normalized by the corresponding internal standard peak area (Equation 1). With the normalized peak area, the affinity value was determined by calculating the fold enrichment of the CDD compounds in the PDCL2 incubated sample over the parallel NTC sample (Equation 2). Among the three tri-synthon compounds (**CDD-1835**, **CDD-1923**, and **CDD-2364**), only the phenanthridinone **CDD-2364** showed a 10-fold enrichment in the PDCL2 incubated sample over the corresponding NTC sample. To study the SAR of the C2 structure in **CDD-2364**, we prepared **CDD-2377** as the truncated version (Scheme 1B), and the affinity value of **CDD-2377** was decreased by 3-fold relative to **CDD-2364** (Figure 4B). The binding affinity of **CDD-2364** and **CDD-2377** was further confirmed in a thermal shift assay (TSA), and both compounds gave an approximately one-degree stabilizing effect to the PDCL2 protein.


(1) $$\text{Normalized peak area of each sample}=\frac{\text{CDD compound peak area}}{\text{Internal standard peak area}}$$

(2) $$\text{Affinity value of CDD compounds}\;\;=\frac{\text{Normalized peak area in the PDCL2 incubated sample}}{\text{Normalized peak area in NTC}}$$

## 3 ∣ CONCLUSIONS

In conclusion, we have screened PDCL2 with a DECL collection containing >440 billion on-DNA compounds, generated three rational structures to perform organic synthesis, synthesized, and characterized four small molecule compounds, and identified two promising compounds (**CDD-2364** and **CDD-2377**) showing binding affinities in our ASMS assay. The ASMS results of the two compounds were further validated by TSA, warranting further SAR studies featuring analogs with modifications on the C2 portion of the **CDD-2364** structure. The functional effects of the optimized lead compounds will be tested in wildtype mice and our recently generated *Pdcl2* knockout mouse model (also being published as part of this special issue).^32^

The flexibility of our DECL and ASMS screening strategy could pave the way for the systematic studies of other nonenzymatic targets, which are challenging due to the lack of biochemical assays. Furthermore, the current work is expected to foster future utilization of the sensitivity and reproducibility of the LC-MS/MS technique to perform semi-quantification ASMS experiments for apparent inhibition constant (K_i,app_) evaluation.

## Supplementary Material

Supplemental

## Figures and Tables

**TABLE 1:** DECL enrichment results for PDCL2 at three different concentrations. Three hit candidates with the same Cycle 1 building block were selected from a tri-synthon DECL library (DEL 1113). The building blocks for cycles 1, 2, and 3 are presented in blue, red, and black, respectively

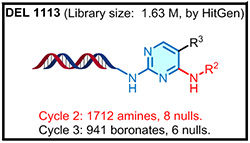			
Cycle 2 building block	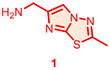	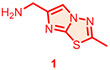	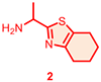
Cycle 3 building block	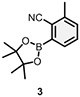	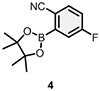	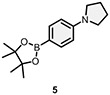
Sequence count at 5 μM	205	72	7
Sequence count at 1 μM	118	52	0
Sequence count at 0.2 μM	14	6	0
Sequence count in blank (0 μM)	0	0	0
