# Supplementary material for: Advancing ASMS with LC-MS/MS for the discovery of novel PDCL2 ligands from DNA-encoded chemical library selections
Source: Andrology. Author manuscript; Available in PMC 2024 Aug 5. (PMC11299427; doi:10.1111/andr.13309)
Supplement: Supplemental [file NIHMS2011220-supplement-Supplemental.docx]

Supporting Information

Advancing ASMS with LC-MS/MS for the Discovery of Novel PDCL2 Inhibitors from DNA-Encoded Chemical Library Selection

Qiuji Ye [^1^](#_bookmark0), Hassane Belabed [^1^](#_bookmark0)^†^, Yong Wang [^1^](#_bookmark0)^†^, Zhifeng Yu [^1^](#_bookmark0)^†^, Murugesan Palaniappan [^1^](#_bookmark0), Jian-Yuan Li [^1^](#_bookmark0), Stacey A. Kalovidouris ^1^, Kevin R. MacKenzie ^1,2^, Mingxing Teng ^1,^[^2^](#_bookmark0), Damian W. Young [^1^](#_bookmark0)^,2^, Yoshitaka Fujihara ^1, 3 *^, and Martin M. Matzuk ^[1](#_bookmark0), 2 *^

^1.^ Center for Drug Discovery, Department of Pathology & Immunology, Baylor College of Medicine, Houston, TX 77030, USA

^2.^ Department of Pharmacology and Chemical Biology, Baylor College of Medicine, Houston, TX 77030, USA

^3.^ National Cerebral and Cardiovascular Center, 6-1 Kishibe-Shimmachi, Suita, Osaka, 564-8565, Japan

Table of Contents

I. Production of Recombinant Protein......................................................................................S2

II. Assay Methods.....................................................................................................................S3

III. General Chemistry Methods...............................................................................................S4

IV. Experimental Procedures and Physical Data of Compounds.............................................S5

V. ^1^H, ^13^C{^1^H}, and HMBC NMR Spectra; IR Spectra of Compounds...................................S11

VI. TSA results for **CDD-2364** and **CDD-2377**……………………………………………S14

**I. Production of Recombinant Protein**

**Purification of human His-PDCL2 recombinant protein.** The N-terminal polyhistidine tag was chosen to construct human PDCL2 (NP_689614.2, amino acid 88-205) in a pET bacterial expression vector for the downstream DEL selection. The cDNA encoding human PDCL2 was synthesized by Genewiz with codon optimization. Sanger sequencing confirmed PDCL2 construct was transformed into E. coli NiCo21 (DE3) cells. Cell colonies were cultured overnight at 37 °C in Luria-Bertani medium (LB-broth, Merck) with 100 µg/mL carbenicillin. These cells were diluted 1:50 in fresh medium and cultured at 37 °C until the optical density reached about 0.7 (OD600). Cells were cooled down to 18 °C before the addition of 0.2 mM isopropyl-*β*-D-thiogalactopyranoside (IPTG) to induce the protein production. After overnight culture, the bacterial cells were harvested by centrifugation at 4,000 rpm for 45 min at 4 °C. Cell pellets were resuspended in lysis buffer containing 50 mM Hepes pH 7.5, 500 mM NaCl, 1 mM TCEP [tris(2-carboxyethyl)phosphinehydrochloride)] and 10 mM imidazole, supplemented with Roche Complete™ Protease Inhibitor Cocktail. The well-resuspended cells were lysed using French Press in a pre-chilled cell. The lysate was cleared by centrifugation at 16,000 g for 1 h at 4 °C on a Beckman Coulter ultracentrifuge and incubated with activated Talon cobalt resins (Takara Bio, USA) overnight at 4 °C with end over end rotation. Talon cobalt resins with protein bound were packed onto a 10 mL glass column to enable a gravity-flow purification of His-tagged PDCL2. The column was washed once with 30 mL of lysis buffer then twice with 10 mL of lysis buffer containing 30 mM imidazole. The protein was eluted using a step elution of imidazole in lysis buffer containing imidazole at 50, 100, 150 and 250 mM. All fractions were collected and monitored by SDS-polyacrylamide gel electrophoresis (Invitrogen NuPAGE™ 4 to 12%, Bis-Tris gradient gel). Fractions with enriched His-tagged PDCL2 were pooled and further purified with size exclusion chromatography on a superdex75 increase 10/300 GL gel filtration column (GE/Amersham Biosciences, USA) at a flow rate of 0.5 mL/min. Samples were monitored by SDS-polyacrylamide gel electrophoresis, pooled, concentrated in the gel filtration buffer (50 mM Hepes pH 7.5, 150 mM NaCl, 1 mM TCEP), aliquoted, flash frozen in liquid nitrogen and stored at −80 °C.

**II. Assay Methods**

**ASMS assay.** The CDD off-DNA compounds (10 mM in DMSO) were diluted into DMSO with a final concentration of 1 mM to make the stock solution. Each CDD compound was further diluted with selection buffer, supplemented with the target protein, and homogenized into a 75 µL solution with 1 µM of CDD compound and 0.5 µM of the target protein. The NTC, and the reference sample was prepared by diluting the 1 mM CDD compound stock solution into a 75 µL solution with selection buffer at 1 µM (NTC), and 0.5 µM (reference) respectively. The target protein containing sample as well as the NTC sample was incubated at 25 °C for 45 min and then subjected to SEC separation according to manufacturer instruction (89808, Thermo Fisher Scientific). The eluent from SEC separation was heated at 80 °C with 1000x shaking for 10 min, diluted with MeOH into a volume of 150 µL, and centrifuged at 10000 rpm for 5 min to obtained 126 µL of the supernatant. The 75 µL of the reference was further diluted into 150 µL with MeOH and saved 126 µL for the following steps. All the samples including the target protein containing sample, the NTC sample, and the reference sample were supplemented with 4 µL of the internal standard (N-methylnaphthalene-1-carboxamide, 20 µM in DMSO).

Liquid chromatography-MS/MS analysis was performed by a Thermo Fisher Scientific TSQ Altis Plus Mass Spectrometer coupled with a 100 mm × 2.1 mm column (XDB C-18, Agilent Technologies). The column temperature was maintained at 40 °C. The flow rate of was 0.5 mL/min with a gradient ranging from 5% to 95% aqueous acetonitrile containing 0.1% formic acid in a 6-min run. The amount of CDD compound was calculated by peak intensity.

**Thermal shift assay.** The dye SYPRO Orange (ThermoFisher Scientific, USA) was used to perform the protein thermal shift assay. The assay was set up on a 384-well Roche plate where His-PDCL2 at a concentration of 2 μM was incubated with the test compound at various concentrations, and SYPRO Orange dye at 5 × in a 10-μL reaction. The melting curve experiment and data analysis was run on a Roche Lightcycler 480 real-time PCR instrument. The TSA was measured two times for each compound.

**III. General Chemistry Information**

All reactions were carried out under an argon atmosphere with dry solvent under anhydrous conditions, unless otherwise noted. Anhydrous acetonitrile (MeCN), acetone, chloroform (CHCl_3_), methanol (MeOH), ethyl acetate (EtOAc) were purchased from commercial suppliers and stored under argon. Yields refer to chromatographically and spectroscopically (^1^H NMR) homogenous material, unless otherwise stated. Reagents were purchased at the highest commercial quality and used without further purification, unless otherwise noted. Reactions were monitored by thin-layer chromatography (TLC) carried out on S-2 0.25 mm E. Merck silica gel plates (60 F_254_) using UV light for visualization and/or an ethanolic solution of phosphomolybdic acid, an aqueous solution of cerium sulfate or a basic aqueous solution of potassium permanganate as staining agents. Luknova SuperSep™ Flash Columns were used for flash column chromatography. NMR spectra were recorded on Bruker DRX-600 or Bruker DRX-800 instrument and calibrated using the resonance signal of the residual undeuterated solvent for ^1^H NMR [δ_H_ =7.26ppm (CDCl_3_), δ_H_ =2.04ppm (CD_3_COOD), and δH =3.31ppm (CD_3_OD)] and deuterated solvent for ^13^C NMR [δ_C_ =77.16 (CDCl_3_), δ_C_ =20.0ppm (CD_3_COOD), and δ_C_ =49.00ppm (CD_3_OD)] as an internal reference at 298 K. The following abbreviations were used to designate multiplicities: s=singlet, d=doublet, t=triplet, q=quartet, quint=quintet, m=multiplet, br=broad. Infrared (IR) spectra were recorded on a PerkinElmer 100 FT-IR spectrometer. High-resolution mass spectra (HRMS) were recorded on a ThermoFisher Scientific Q Exactive Orbitrap Mass Spectrometer using ESI (electrospray ionization).

**IV. Experimental Procedures and Characterization Data**

**{(3-Methyl-4-thia-1,2,6-triazabicyclo[3.3.0]octa-2,5,7-trien-7-yl)methyl}(5-bromo-2-chloro-4-pyrimidinyl)amine (7a):** To a stirred solution of the dichloropyrimidine **6** (456 mg, 2.0 mmol, 1.0 equiv) in CH_3_CN (4 mL) at 23 °C was added the amine **1** (336 mg, 2.0 mmol, 1.0 equiv) and Et_3_N (404 mg, 4.0 mmol, 2.0 equiv). The resulting mixture was refluxed for 18 h. The reaction mixture was cooled to room temperature, evaporated to dryness under reduced pressure, and the obtained residue was purified by flash column chromatography (SiO_2_, 0→5% CH_3_OH in CH_2_Cl_2_) to afford **7a** (230 mg, 0.64 mmol, 32% yield) as colorless solid. ^1^HNMR (600 MHz, CDCl_3_) δ ppm 8.06 (s, 1H), 7.65 (s, 1H), 6.23 (s, 1H), 4.64 (d, *J* = 5.4 Hz, 2H), 2.64 (s, 3H).

**[1-(4,5,6,7-Tetrahydro-1-thia-3-aza-2-indenyl)ethyl](5-bromo-2-chloro-4-pyrimidinyl)amine (7b):** To a stirred solution of the dichloropyrimidine **6** (456 mg, 2.0 mmol, 1.0 equiv) in CH_3_CN (4 mL) at 23 °C was added the amine **2** (364 mg, 2.0 mmol, 1.0 equiv) and Et_3_N (404 mg, 4.0 mmol, 2.0 equiv). The resulting mixture was refluxed for 18 h. The reaction mixture was cooled to room temperature, evaporated to dryness under reduced pressure, and the obtained residue was purified by flash column chromatography (SiO_2_, 0→5% CH_3_OH in CH_2_Cl_2_) to afford **7b** (254 mg, 0.68 mmol, 34% yield) as colorless solid. ^1^HNMR (600 MHz, CDCl_3_) δ ppm 8.16 (s, 1H), 6.47 (s, 1H), 5.56 (m, 1H), 2.77 (m, 4H), 1.86 (m, 4H), 1.7 (d, *J* = 6.6 Hz, 3H).

**5-Bromo-2-(methylamino)-4-({(3-methyl-4-thia-1,2,6-triazabicyclo[3.3.0]octa-2,5,7-trien-7-yl)methyl}amino)pyrimidine (8a):** To a stirred solution of the chloropyrimidine **7a** (50 mg, 0.14 mmol, 1.0 equiv) in EtOH (3 mL) at 23 °C was added the CH_3_NH (2.0 ᴍ in THF, 210 µL, 0.42 mmol, 3.0 equiv). The resulting mixture was heated at 110 °C for 18 h. The reaction mixture was cooled to room temperature, evaporated to dryness under reduced pressure, and the obtained residue was purified by flash column chromatography (SiO_2_, 0→5% CH_3_OH in CH_2_Cl_2_) to afford **8a** (32 mg, 0.09 mmol, 64% yield) as colorless solid. ^1^H NMR (600 MHz, CDCl_3_, mixture of tautomers) δ 7.86 (s, 1H), 7.61 (s, 1H), 5.78 (s, 0.5H), 5.08 (s, 0.5H), 4.64 (s, 2H), 2.92 (s, 3H), 2.66 (s, 3H). ^13^C NMR (151 MHz, CDCl_3_, mixture of tautomers) δ 161.5, 161.5, 159.3, 158.0, 157.9, 155.6, 144.9, 143.5, 111.2, 39.4, 39.2, 28.5, 28.4, 17.8. HRMS (ESI-orbitrap) *m/z*: Calcd. for C_11_H_13_BrN_7_S^+^ 354.0132; Found 354.0136.

**5-Bromo-2-(methylamino)-4-[1-(4,5,6,7-tetrahydro-1-thia-3-aza-2-indenyl)ethylamino]pyrimidine (8b):** To a stirred solution of the chloropyrimidine **7b** (15 mg, 0.040 mmol, 1.0 equiv) in EtOH (1 mL) at 23 °C was added the CH_3_NH (2.0 ᴍ in THF, 60 µL, 0.12 mmol, 3.0 equiv). The resulting mixture was heated at 110 °C for 18 h. The reaction mixture was cooled to room temperature, evaporated to dryness under reduced pressure, and the obtained residue was purified by flash column chromatography (SiO_2_, 0→5% CH_3_OH in CH_2_Cl_2_) to afford **8b** (5 mg, 0.013 mmol, 34% yield) as colorless solid. ^1^H NMR (600 MHz, CDCl_3_, mixture of tautomers) δ 7.85 (s, 1H), 5.77 (d, *J* = 6.6 Hz, 1H), 5.52 (m, 1H), 5.43 – 5.23 (m, 1H), 3.41 (s, 1H), 3.35 (s, 1H), 2.85 (d, *J* = 5.4 Hz, 3H), 2.69 (m, 4H), 1.79 (m, 4H), 1.64 (d, *J* = 7.2 Hz, 3H). ^13^C NMR (151 MHz, CDCl_3_, mixture of tautomers) δ 169.4, 161.4, 157.3, 156.0, 149.9, 50.0, 48.3, 28.5, 26.7, 23.4, 23.3, 22.9, 21.7. HRMS (ESI-orbitrap) *m/z*: Calcd. for C_14_H_15_BrN_5_S^+^ 368.0540; Found 368.0550.

**3-[2-(Methylamino)-4-({(3-methyl-4-thia-1,6-diazabicyclo[3.3.0]octa-2,5,7-trien-7-yl)methyl}amino)-5-pyrimidinyl]-2-toluonitrile (CDD-1923):** To a microwave reaction vial at 23 °C were added 8a (10 mg, 0.028 mmol, 1.0 equiv), 3 (8.3 mg, 0.034 mmol, 1.2 equiv), K_3_PO_4_ (12 mg, 0.056 mmol, 2.0 equiv), cataCXium^®^ A (Sigma-Adrich^®^, 1.1 mg, 0.003 mmol, 0.1 equiv), Pd_2_(dba)_3_ (1.4 mg, 0.0015 mmol, 0.05 equiv), followed by dioxane (2.0 mL) and H_2_O (0.5 mL). The vial was then sealed and irrediated in a microwave reactor (Biotage: Initiator) to a temperature of 110 °C for 30 min. cooled to room temperature, evaporated to dryness under reduced pressure, and the obtained residue was purified by C-18 flash column chromatography (Si-(CH_2_)_17_-CH_3_, 0→100% CH_3_OH in H_2_O) to afford **CDD-1923** (7.2 mg, 0.018 mmol, 64% yield) as colorless solid. ^1^HNMR (600 MHz, CDCl_3_) δ ppm 8.99 (bs, 1H), 7.93 (d, *J* = 7.8 Hz, 1H), 7.69 (s, 1H), 7.56 (t, *J* = 8.4 Hz, 1H), 7.27-7.25 (m, 2H), 5.72 (bs, 2H), 3.12 (d, *J* = 4.6 Hz, 3H), 2.93 (s, 3H), 2.66 (s, 3H). ^13^C NMR (151 MHz, CDCl_3_) δ ppm 163.2, 159.4, 159.3, 144.3, 143.3, 142.6, 142.5, 139.7, 133.2, 132.4, 130.8, 126.2, 123.7, 122.1, 117.5, 39.0, 28.6, 24.4, 17.7. HRMS (ESI-orbitrap) *m/z*: Calcd. for C_19_H_19_N_8_S^+^ 391.1448; Found 391.1444. No thermal shift was observed for this compound in TSA assay.

**2-(Methylamino)-5-[*p*-(1-pyrrolidinyl)phenyl]-4-[1-(4,5,6,7-tetrahydro-1-thia-3-aza-2-indenyl)ethylamino]pyrimidine (CDD-1835):** To a microwave reaction vial at 23 °C were added 8b (10 mg, 0.027 mmol, 1.0 equiv), 5 (6.1 mg, 0.032 mmol, 1.2 equiv), K_3_PO_4_ (11.6 mg, 0.054 mmol, 2.0 equiv), cataCXium^®^ A (Sigma-Adrich^®^, 1.1 mg, 0.003 mmol, 0.1 equiv), Pd_2_(dba)_3_ (1.4 mg, 0.0015 mmol, 0.05 equiv), followed by dioxane (2.0 mL) and H_2_O (0.5 mL). The vial was then sealed and irrediated in a microwave reactor (Biotage: Initiator) to a temperature of 110 °C for 30 min. cooled to room temperature, evaporated to dryness under reduced pressure, and the obtained residue was purified by C-18 flash column chromatography (Si-(CH_2_)_17_-CH_3_, 0→100% CH_3_OH in H_2_O) to afford **CDD-1835** (8.0 mg, 0.018 mmol, 68% yield) as colorless solid. ^1^HNMR (600 MHz, CDCl_3_) δ ppm 7.60 (s, 1H), 7.13 (d, *J* = 8.8 Hz, 2H), 6.57, (d, *J* = 8.8 Hz, 2H), 5.55 (m, 2H), 3.25 (m, 4H), 2.90 (d, *J* = 4.8 Hz, 3H), 2.67 (m, 4H), 1.96 (m, 4H), 1.77 (m, 4H), 1.50 (d, *J* = 6.0 Hz, 3H). ^13^C NMR (151 MHz, CDCl_3_) δ ppm 170.6, 161.0, 159.5, 153.1, 149.9, 147.4, 129.9, 128.2, 121.0, 112.3, 112.2, 110.6, 53.4, 48.1, 47.6, 28.4, 26.8, 25.5, 23.5, 23.4, 23.0, 21.7. HRMS (ESI-orbitrap) *m/z*: Calcd. for C_24_H_31_N_6_S^+^ 435.2326; Found 435.2317. No thermal shift was observed for this compound in TSA assay.

**6-Fluoro-2-(methylamino)-10-{(3-methyl-4-thia-1,2,6-triazabicyclo[3.3.0]octa-2,5,7-trien-7-yl)methyl}-9,10-dihydro-1,3,10-triaza-9-phenanthrenone (CDD-2364):** To a microwave reaction vial at 23 °C were added 8a (10 mg, 0.028 mmol, 1.0 equiv), 4 (8.4 mg, 0.034 mmol, 1.2 equiv), K_3_PO_4_ (12 mg, 0.056 mmol, 2.0 equiv), cataCXium^®^ A (Sigma-Adrich^®^, 1.1 mg, 0.003 mmol, 0.1 equiv), Pd_2_(dba)_3_ (1.4 mg, 0.0015 mmol, 0.05 equiv), followed by dioxane (2.0 mL) and H_2_O (0.5 mL). The vial was then sealed and irrediated in a microwave reactor (Biotage: Initiator) to a temperature of 110 °C for 30 min. cooled to room temperature, evaporated to dryness under reduced pressure, and the obtained residue was purified by C-18 flash column chromatography (Si-(CH_2_)_17_-CH_3_, 0→100% CH_3_OH in H_2_O) to afford **CDD-2364** (8.5 mg, 0.021 mmol, 77% yield) as colorless solid. ^1^H NMR (800 MHz, acetic acid-*d*_4_) δ 9.14 (s, 1H), 8.47 (m, 1H), 7.96 – 7.83 (m, 2H), 7.32 (t, *J* = 7.5 Hz, 1H), 5.78 (s, 2H), 3.11 (s, 3H), 2.68 (s, 3H). ^13^C NMR (201 MHz, acetic acid-*d*_4_) δ 166.4 (d, *J* = 253.5 Hz), 162.7, 162.4, 157.8, 156.3, 148.7, 144.9, 139.9, 134.1 (d, *J* = 11.0 Hz), 132.2 (d, *J* = 10.2 Hz), 120.0, 115.9 (d, *J* = 23.2 Hz), 112.7, 106.5 (d, *J* = 23.9 Hz), 102.9, 38.5, 27.5, 16.3. HRMS (ESI-orbitrap) *m/z*: Calcd. for C_18_H_15_FN_7_OS^+^ 396.1037; Found 396.1034.

**5-Bromo-2,4-bis(methylamino)pyrimidine (10):** To a stirred solution of the dichloropyrimidine **6** (456 mg, 2.0 mmol, 1.0 equiv) in DIPEA:DMA (2:1, *v*/*v*, 4 mL) at 23 °C was added MeNH_2_•HCl (804 mg, 12 mmol, 6.0 equiv). The resulting mixture was heated at 110 °C for 18 h. The reaction mixture was cooled to room temperature, evaporated to dryness under reduced pressure, and the obtained residue was purified by C-18 flash column chromatography (Si-(CH_2_)_17_-CH_3_, 0→100% CH_3_OH in H_2_O) to afford **10** (304 mg, 1.4 mmol, 70% yield) as colorless solid. ^1^H NMR (600 MHz, methanol-*d*_4_) δ 7.73 (s, 1H), 2.94 (s, 3H), 2.87 (s, 3H). ^13^C NMR (151 MHz, methanol-*d*_4_) δ 161.5, 159.1, 155.2, 154.1, 27.2, 26.6. HRMS (ESI-orbitrap) *m/z*: Calcd. for C_6_H_10_BrN_4_^+^ 217.0084; Found 217.0086.

**9-Fluoro-5-methyl-3-(methylamino)pyrimido[4,5-*c*]isoquinolin-6(5*H*)-one (CDD-2377):** To a microwave reaction vial at 23 °C were added **10** (22 mg, 0.10 mmol, 1.0 equiv), **4** (29.6 mg, 0.12 mmol, 1.2 equiv), K_3_PO_4_ (42 mg, 0.2 mmol, 2.0 equiv), cataCXium^®^ A (Sigma-Adrich^®^, 3.6 mg, 0.01 mmol, 0.1 equiv), Pd_2_(dba)_3_ (4.6 mg, 0.005 mmol, 0.05 equiv), followed by dioxane (2.4 mL) and H_2_O (0.6 mL). The vial was then sealed and irrediated in a microwave reactor (Biotage: Initiator) to a temperature of 110 °C for 30 min. cooled to room temperature, evaporated to dryness under reduced pressure, and the obtained residue was purified by C-18 flash column chromatography (Si-(CH_2_)_17_-CH_3_, 0→100% CH_3_OH in H_2_O) to afford **CDD-2377** (18.6 mg, 0.07 mmol, 72% yield) as colorless solid. ^1^H NMR (600 MHz, acetic acid-*d*_4_) δ 8.91 (s, 1H), 8.27 (dd, *J* = 8.8, 5.8 Hz, 1H), 7.66 (dd, *J* = 9.8, 2.0 Hz, 2H), 7.14 (d, *J* = 16.8 Hz, 1H), 3.59 (s, 3H), 2.95 (s, 2H). ^13^C NMR (151 MHz, acetic acid-*d*_4_) δ 167.4 (d, *J* = 253.3 Hz), 163.8, 159.1, 157.7, 149.7, 134.9 (d, *J* = 10.2 Hz), 133.2 (d, *J* = 10.3 Hz), 120.9 (d, *J* = 1.3 Hz), 116.9 (d, *J* = 23.3 Hz), 107.4 (d, *J* = 24.3 Hz), 103.7, 28.9, 28.4. HRMS (ESI-orbitrap) *m/z*: Calcd. for C_13_H_12_FN_4_O^+^ 259.0990; Found 259.0989.

**V. ^1^H, ^13^C{^1^H}, and HMBC NMR Spectra; IR Spectra of CDD-2364**

MeOH

HMBC data of CDD-2377 shown the correlation between C-10 with H-18, C-10 with H-13, and C-10 with H-16, indicating the phenanthrenone core is formed in the product structure.

**VI. TSA results for CDD-2364 and CDD-2377**
